# To Make a Duplicate of a Denture

**Published:** 1899-02

**Authors:** Ellison Hillyer

**Affiliations:** the New York College of Dentistry.


### ARTICLE IX.

### TO MAKE A DUPLICATE OF A DENTURE.

DR. ELLISON HILLYER,

Of the New York College of Dentistry.

To make an exact duplicate of a full denture of vulcanite.

1. Cleanse thoroughly.

2. Cast the model upon the palatal portion of the plate, not necessarily extending posterior to the condyles—(oiling the plate is unnecessary).

3. To duplicate the "fullness" of the lateral portions and the mold and size of the teeth, cast two sections of plaster entending (a) from condyle to median line, and (b) from median line to opposite condyle, and from the base of the model to the cutting-edge of the teeth. When plaster comes in contact with plaster, Oliver's separating fluid—or other—may be used.

4. To duplicate the exact thickness of the denture, cast the lingual portion, extending from condyle to condyle and covering the lingual serface to the edge of the cast, labial sections (a and b) extending thus over the cutting-edges of the teeth.

With these four parts "set" and removed, the teeth may be selected to fit the labial molds—proper shade—and ground exactly to fit, then set up and waxed upon the model—the labial models being in position relative to the model—the thickness of the wax determined by the lingual cast in its proper relative position. When vulcanized and finished, an exact duplicate should result.

Dr. Wilbur F. Litch, of the Pennsylvania College of Dental Surgery, says that to make an exact duplicate of a full denture, either with or without the patient, would be practically impossible. To produce an approximately exact duplicate in the absence of the patient, run plaster into the plate to make a new cast. If there should be so much undercut that the cast cannot be separated from the plate without breaking, run the cast in sections, then to be firmly joined together for working purposes.

Placing upon the cast thus obtained the denture to be reproduced, run outside of it and the cast a plaster matrix in two or more sections to serve as guides for fullness and contour, length and position of the teeth.

Make the "bite" of a fusible alloy melting at about the temperature of boiling water; this can be run upon the grinding and cutting-edges of the teeth without danger of fracturing them, and mounted in a suitable articulating frame—Bonwill's preferably—can then be used for mounting the teeth as would the ordinary plaster bite, with the advantage that it can not readily be broken, and will not rapidly wear away in the prolonged grinding which would be required to procure an exact reproduction of the grinding and incisive edges of the denture to be duplicated. Teeth made in the same molds as the originals would probably be necessary.

Dr. Robert H. Nones, of the Medico-Chirurgical College, of Philadelphia, maintains that it is not possible to make an exact duplicate of a denture, from the fact that the contraction and expansion of materials used in cast, die, and plate all operate to make slight changes; however, assuming that it is a denture mounted upon vulcanite to be duplicated.

1. Wash and scrub the plate with dilute ammonia water to free it from grease and other foreign material.

2. Slightly, very slightly, oil all of the surfaces of plate and teeth with olive oil.

3. Pour a cast in the palatal portion of the plate one-half inch thick, or more, and have it project beyond the edges of the plate all around. Smooth this cast and varnish its outer walls, in which three or more conical depressions are made above the plate-rim edges.

Remove plate from cast and set it back. Form a plaster wall over one-quarter inch thick about the outer sides of dentures and outer wall of cast. Place cast and all in an articulator, and run a plaster mold over plate and occlusal turfaces of teeth. Lock the articulator jaws firmly. When the plaster is hard, separate jaws of articulator, pry oft the outer plaster walls. Select a set of teeth exactly like the others, and set them in the outer plaster walls. Make a base-plate of wax, put walls and teeth in position, and press softened wax beneath teeth until they are firmly held. Add more wax, faintly oil the plaster mold of the occlusal edges, and press the articulator jaws in position. See that each tooth fits exactly into the two plaster guides. Next, finish waxing, invest, pack, vulcanite and finish. The inevitable shrinking of the vulcanite will make the second plate slightly different from the first plate.

Dr. George H. Wilson, of the Western Reserve University, Cleveland, Ohio, says that after giving the plate and teeth a thin coat of oil, fill, making the plaster cast. With the plate upon the cast, attach to one jaw of the articulator; set the other jaw of the articulator about one-fourth inch from the teeth; mix plastar stiff enough to stand without flowing, spread upon the occlusal and incisive ends of the teeth, overlapping the buccal, labial and lingual surfaces about one-sixteenth of an inch; place the articulator in position and unite with plaster. When the plaster has hardened, open the articulator and remove the denture; place base-plate wax upon the cast, with a roll of wax upon the alveolar ridge; set duplicate teeth in imprints of teeth upon opposite jaw of articulator, close articulator, forcing the cervical ends of the teeth into the wax roll; with hot spatula unite teeth and wax roll to base-plate. After cooling the wax, trim the plaster away from the buccal and labial surfaces of the teeth; open the articulator and build the wax plate to contour. Now remove the lingual portion of the plaster imprint of the bicuspids and molars; close the articulator, proving the articulation. Now the case will receive the ordinary manipulations.

If the case is a metal denture, it will be necassary to make two cases, one to be used as a model in producing the die.

Dr. R. R. Freeman, of Vanderbilt University; gets accurate casts from upper and lower plates. With plates on casts, occlude and adjust according to the Bonwill rule, on a Bonwill articulator. Remove plates, run additional casts sufficiently thick and slightly expanded at base for countersinks; place each on level plane, plates on casts. Invest teeth in plaster, two sections, extending from plane up over teeth, dividing at median line. Having soaped your plaster will separate readily where desired.

Separate sections, remove plates, adjust base-plate of wax on cast, and replace sections. If you have duplicate teeth, they may be readily adjusted to position in teeth impression left in sections; otherwise make best selections you can and wax up. Remove these wax plates with teeth arranged to the casts in articulator for more accurate occlusion.

Return to your duplicate casts, wax to a finish, trim casts, flask, etc.; after vulcanizing and finishing up plates, to be certain beyond question that your occlusion is same as original plates, take casts from duplicate plates. on these casts adjust wax plates, transfer to original casts in articulator, build up at right length, fasten wax plates together, and in these place the duplicate casts, and adjust by Bonwill rule in Bonwill articulator.

Remove the wax base-plates, substitute the duplicate plates and do such grinding as may be necessary to perfect occlusion, and you will have an exact duplicate of your full denture.—*Cosmos.*

